# Estimation of country-specific and global prevalence of male circumcision

**DOI:** 10.1186/s12963-016-0073-5

**Published:** 2016-03-01

**Authors:** Brian J Morris, Richard G Wamai, Esther B Henebeng, Aaron AR Tobian, Jeffrey D Klausner, Joya Banerjee, Catherine A Hankins

**Affiliations:** School of Medical Sciences and Bosch Institute, University of Sydney, Sydney, NSW 2006 Australia; Department of African-American Studies, Northeastern University, Boston, MA 02115 USA; College of Science, Northeastern University, Boston, MA 02115 USA; Department of Pathology, School of Medicine, Johns Hopkins University, Baltimore, MD 21287 USA; Division of Infectious Diseases and Program in Global Health, David Geffen School of Medicine, University of California Los Angeles (UCLA), Los Angeles, CA 90095 USA; Jhpiego, an affiliate of Johns Hopkins University, Washington, DC 20009 USA; Department of Global Health, Academic Medical Centre and Amsterdam Institute for Global Health and Development, University of Amsterdam, Amsterdam, 1105 AZ The Netherlands; Department of Infectious Disease Epidemiology, Faculty of Epidemiology and Population Health, London School of Hygiene and Tropical Medicine, London, WC1E 7HT UK

**Keywords:** Male circumcision, Global prevalence, Country-specific prevalence, Population health

## Abstract

**Background:**

Male circumcision (MC) status and genital infection risk are interlinked and MC is now part of HIV prevention programs worldwide. Current MC prevalence is not known for all countries globally. Our aim was to provide estimates for country-specific and global MC prevalence.

**Methods:**

MC prevalence data were obtained by searches in PubMed, Demographic and Health Surveys, AIDS Indicator Surveys, and Behavioural Surveillance Surveys. Male age was ≥15 years in most surveys. Where no data were available, the population proportion whose religious faith or culture requires MC was used. The total number of circumcised males in each country and territory was calculated using figures for total males from (i) 2015 US Central Intelligence Agency (CIA) data for sex ratio and total population in all 237 countries and territories globally and (ii) 2015 United Nations (UN) figures for males aged 15–64 years.

**Results:**

The estimated percentage of circumcised males in each country and territory varies considerably. Based on (i) and (ii) above, global MC prevalence was 38.7 % (95 % confidence interval [CI]: 33.4, 43.9) and 36.7 % (95 % CI: 31.4, 42.0). Approximately half of circumcisions were for religious and cultural reasons. For countries lacking data we assumed 99.9 % of Muslims and Jews were circumcised. If actual prevalence in religious groups was lower, then MC prevalence in those countries would be lower. On the other hand, we assumed a minimum prevalence of 0.1 % related to MC for medical reasons. This may be too low, thereby underestimating MC prevalence in some countries.

**Conclusions:**

The present study provides the most accurate estimate to date of MC prevalence in each country and territory in the world. We estimate that 37–39 % of men globally are circumcised. Considering the health benefits of MC, these data may help guide efforts aimed at the use of voluntary, safe medical MC in disease prevention programs in various countries.

**Electronic supplementary material:**

The online version of this article (doi:10.1186/s12963-016-0073-5) contains supplementary material, which is available to authorized users.

## Background

The circumcision of males is one of the most common surgical procedures in the world. It may also be one of the oldest, likely predating recorded human history [1]. While religious and cultural considerations are a major reason behind the practice, a growing volume of research attests to the significant medical and public health benefits of male circumcision (MC) [2–5]. Health, medical, sexual, and cosmetic benefits may explain why MC is a fundamentally inherent part of diverse human cultures globally, especially in hot arid environments [1]. In light of the protection that MC affords against HIV infection in particular [6], major health bodies such as the World Health Organization (WHO), the Joint United Nations Program on HIV/AIDS (UNAIDS), and the US Centers for Disease Control and Prevention (CDC) have endorsed and currently promote voluntary medical MC (VMMC) in HIV-1 epidemic settings in which the major route of HIV transmission is through heterosexual intercourse [7, 8]. In sub-Saharan African countries, 13 were prioritized by WHO and UNAIDS for VMMC for HIV prevention in 2007, with Ethiopia’s Gambella province, the Central African Republic, and South Sudan added subsequently [9]. Implementation is well underway in the original 13 countries, with over 10 million VMMC performed since 2009 [10].

In 2012, the American Academy of Pediatrics produced an affirmative infant MC policy statement that highlighted the ability of MC to protect against multiple conditions over a lifetime. The report stated that early in pregnancy, parents should be routinely informed of the benefits and the low risk of MC, and that third party payment for MC is warranted [11]. The CDC has determined that the overall adverse event rate for early infant MC in the US is less than 0.5 %, with the rate 10–20 times higher in older boys and men than in neonates, [12] reflecting the advantages of early infant MC over adult MC [13]. Benefits exceed risks many fold, with half of uncircumcised males developing at some point at least one of the conditions against which MC offers either partial or complete lifelong protection [4]. The US has, arguably, the highest quality medical technology and expertise available in the world to those who can afford it. The most common procedure for children in US hospitals is prophylactic vaccination (1,329,600) followed by MC (1,147,700) [14], making MC the most common procedure in boys.

A 2007 WHO Report estimated that approximately 30 % of the world’s males aged 15 years or older were circumcised [15]. In 2011, an estimate by an independent researcher found global MC prevalence to be 37–40 % [16]. After examination of these previous estimates, we considered them to be out of date, since no data were provided for many countries, neither estimate was published in a peer-reviewed journal, and numerous new surveys have appeared since then. We therefore considered it timely to conduct a much more thorough evaluation aimed at determining the latest MC prevalence figures for every country in the world, especially in view of recent policy trends supporting the scale-up of VMMC implementation.

The aim of the present study was to determine, as realistically as possible, (i) current country-by-country and (ii) global prevalence of MC.

## Methods

### Literature searches

**Fig. 1:**
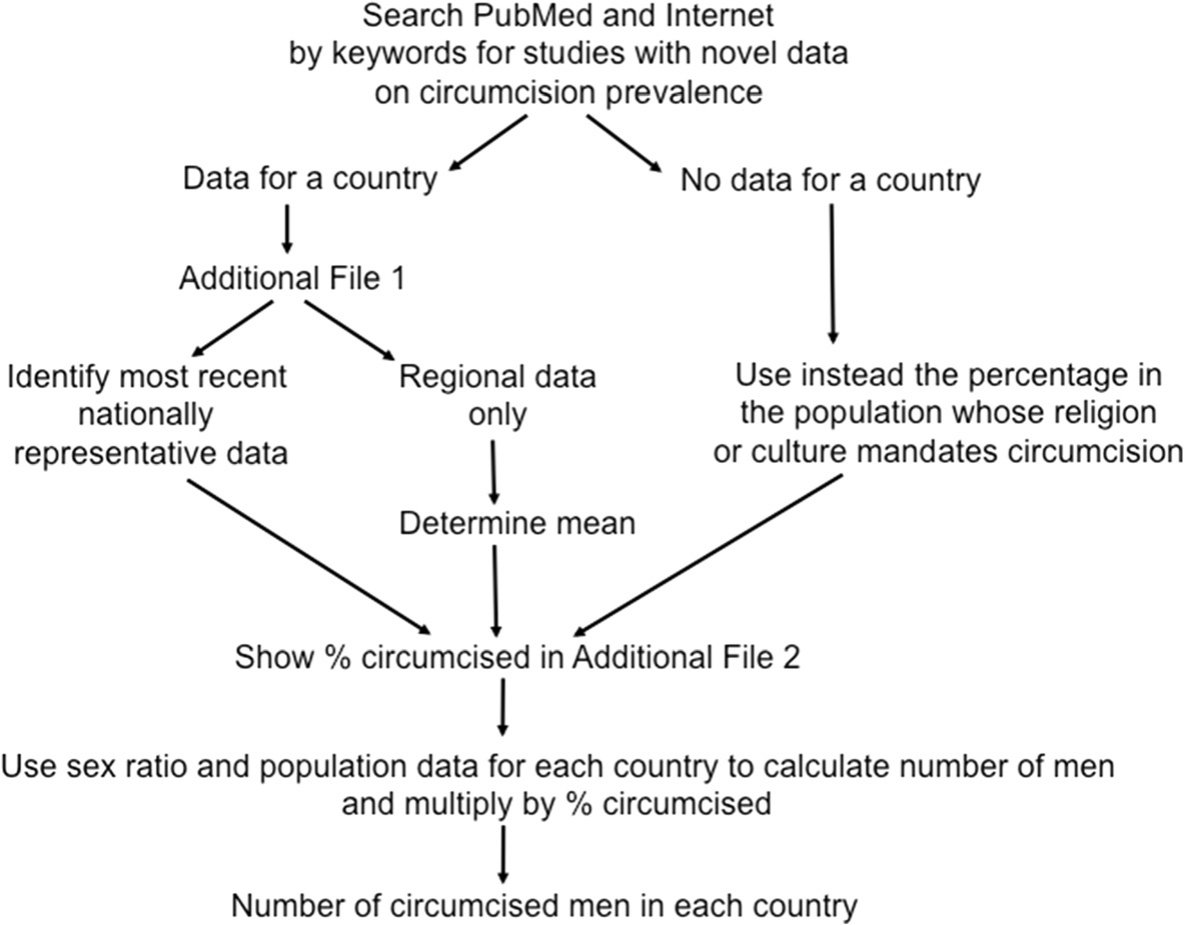
Flow chart of strategy used to obtain an estimate of circumcision prevalence in each country

The method we used to determine MC prevalence in each country is shown in Fig. 1. Prevalence of recorded MC was extracted from published articles retrieved through a PubMed search on 2 June 2014 (updated 1 Oct 2015) using the search terms “male circumcision” combined with either “rate” (480 hits), “prevalence” (1497 hits) or “incidence” (1548 hits). Articles published prior to 2000 were excluded since MC practices can change over time. Preference was given to the most recent nationally-representative surveys, where available, to arrive at the most valid MC prevalence estimate in a country. Available subnational data, such as in Brazil, are shown as well to illustrate variation in MC prevalence between different parts of a country. Altogether, 143 publications contained relevant MC data for inclusion, of which 78 were dated from 2010 onwards.

Since MC takes place at different ages according to varying cultural practices, the sources we included contained data for mature males rather than boys, with the exception of Canada where only data for infants are available. Survey data for mature males therefore included MCs performed for cultural reasons in childhood and adolescence. We obtained data on MC prevalence both in countries in which it is practiced for religious, cultural, and/or health reasons and in countries where personal preference of the male or his parents predominates. Most of the latter are developed Anglophone countries, such as the US, Canada, Australia, and New Zealand.

We performed Internet searches for documents produced by authoritative bodies, including Demographic and Health Surveys (DHS), AIDS Indicator Surveys (AIS), and Behavioural Surveillance Surveys (BSS) in specific countries. Data on MC prevalence were compiled in Table 1 along with pertinent information such as whether the survey was nation-wide or involved a particular locality, the denominator or “n” value on which the estimate was based, and the age range of the males surveyed. Where possible, preference was given to nation-wide data rather than local estimates. When more than one study provided what appeared to be a representative MC prevalence for a particular country, preference was given to larger, most recent surveys. If there was more than one suitable data source, a weighted average was used to estimate percentage of circumcised men in the country.

**Table 1:** Percentage of circumcised males in each of the 237 countries and territories in the world^a^

Country/territory	MC %	Country/territory	MC %	Country/territory	MC %
Afghanistan	99.8	Ghana	91.6	Oman	87.7
Albania	47.7	Gibraltar	6	Pakistan	96.4
Algeria	97.9	Greece	4.7	Palau	95
American Samoa	95	Greenland	0.1	Panama	0.95
Andorra	1.1	Grenada	0.3	Papua New Guinea	10.1
Angola	57.5	Guam	95	Paraguay	0.11
Anguilla	0.3	Guatemala	0.11	Peru	3.7
Antigua & Barbuda	0.6	Guernsey	0.1	Philippines	91.7
Argentina	2.9	Guinea	84.2	Pitcairn Islands	0
Armenia	0.1	Guinea-Bissau	93.3	Poland	0.11
Aruba	0.46	Guyana	12	Portugal	0.61
Australia	26.6	Haiti	6.2	Puerto Rico	0.14
Austria	5.8	Holy See (Vatican)	0.1	Qatar	77.5
Azerbaijan	98.5	Honduras	0.1	Romania	0.34
Bahamas, The	0.2	Hong Kong	28	Russia	11.8
Bahrain	81.2	Hungary	0.78	Rwanda	13.3
Bangladesh	93.2	Iceland	0.1	Saint Barthelemy	0.1
Barbados	0.9	India	13.5	Saint Helena, Ascens	0.1
Belarus	0.32	Indonesia	92.5	Saint Kitts & Nevis	0.3
Belgium	22.6	Iran	99.7	Saint Lucia	0.1
Belize	0.1	Iraq	98.9	Saint Martin & Tristan	0.1
Benin	92.9	Ireland	0.93	Saint Pierre & Miquel	0.2
Bermuda	0.8	Isle of Man	0.2	Saint Vincent & Grena	1.7
Bhutan	1.0	Israel	91.7	Samoa	95
Bolivia	0.11	Italy	2.6	San Marino	0.1
Bosnia & Herzegovina	41.6	Jamaica	14	Sao Tome & Principe	0.1
Botswana	15.1	Japan	9	Saudi Arabia	97.1
Brazil	1.3	Jersey	0.1	Senegal	93.5
British Virgin Islands	1.2	Jordan	98.8	Serbia	3.71
Brunei	51.9	Kazakhstan	56.4	Seychelles	1.1
Bulgaria	13.4	Kenya	91.2	Sierra Leone	96.1
Burkin Faso	88.3	Kiribati	0.1	Singapore	14.9
Burma	3.5	Korea, North	0.1	Sint Maarten	0.06
Burundi	61.7	Korea, South	77.0	Slovakia	0.15
Cabo Verde	0.1	Kosovo Islands	91.7	Slovenia	8.5
Cambodia	3.5	Kuwait	86.4	Solomon Islands	95
Cameroon	94.0	Kyrgyzstan	91.9	Somalia	93.5
Canada	31.9	Laos	0.1	South Africa	44.7
Cayman Islands	0.2	Latvia	0.38	South Sudan	23.6
Central African Republic	63.0	Lebanon	59.7	Spain	6.6
Chad	73.5	Lesotho	52	Sri Lanka	8.5
Chile	0.21	Liberia	97.7	Sudan	39.4
China	14.0	Libya	96.6	Suriname	15.9
Christmas Island	0.1	Liechtenstein	4.8	Svalbard	0.1
Cocos (Keeling)	95	Lithuania	0.2	Swaziland	8.2
Columbia	4.2	Luxembourg	2.4	Sweden	5.1
Comoros	99.4	Macau	0.1	Switzerland	5.9
Congo, Democrat Repub	97.2	Macedonia	33.9	Syria	92.8
Congo, Republic	70	Madagascar	94.7	Taiwan	8.3
Cook Islands	95	Malawi	21.6	Tajikistan	99
Costa Rica	0.15	Malaysia	61.4	Tanzania	72
Cote d’Ivoire	96.7	Maldives	98.4	Thailand	23.4
Croatia	1.3	Mali	86	Timor-Leste	6.4
Cuba	0.11	Malta	0.3	Togo	95.2
Curacao	0.07	Marshall Islands	0.1	Tokelau	95
Cyprus	22.7	Mauritania	99.2	Tonga	95
Czech Republic	0.14	Mauritius	16.6	Trinidad & Tobago	5.8
Denmark	5.3	Mexico	15.4	Tunisia	99.8
Djibouti	96.5	Micronesia, Fed States	0.1	Turkey	98.6
Dominica	0.2	Moldova	0.5	Turkmenistan	93.4
Dominican Republic	13.7	Monaco	0.5	Turks & Caicos Is	0.1
Ecuador	0.11	Mongolia	4.4	Tuvalu	95
Egypt	94.7	Montenegro	18.5	Uganda	26.7
El Salvador	0.11	Montserrat	0.1	Ukraine	2.3
Equatorial Guinea	87	Morocco	99.9	United Arab Emirates	76
Eritrea	97.2	Mozambique	47.4	United Kingdom	20.7
Estonia	0.25	Namibia	25.5	United States	71.2
Ethiopia	92.2	Nauru	95	Uruguay	0.62
Falkland Islands	0.1	Nepal	4.2	Uzbekistan	96.5
Faroe Islands	0.1	Netherlands	5.7	Vanuatu	95
Fiji	55	New Caledonia	50	Venezuela	0.33
Finland	0.82	New Zealand	33.0	Vietnam	0.2
France	14	Nicaragua	0.1	Virgin Islands	0.55
French Polynesia	78	Niger	95.5	Wallis & Futuna	0.1
Gabon	99.2	Nigeria	98.9	West Bank	99.9
Gambia, The	94.5	Niue	95	Western Sahara	99.6
Gaza Strip	99.9	Norfolk Island	0.1	Yemen	99.0
Georgia	10. 6	Northern Mariana Is	90	Zambia	12.8
Germany	10.9	Norway	3.0	Zimbabwe	9.2

^a^The reader is referred to Additional files 1 and 2 in the supplementary material in order to understand the use of our methodology for deriving the values shown in this Table

### Countries lacking data

For many countries, no data were available for MC prevalence. We therefore prepared estimates of MC performed for religious or cultural reasons or medical treatment. MC is virtually universal in Jewish and Muslim populations [1, 15, 17]. Data for Muslim population sizes in various countries usually are known more precisely than MC prevalence in those countries [18, 19]. Data for the percentage of Jews by country were obtained from the Jewish Virtual Library [20]. Data for the percentage of Muslims by country were obtained from Pew Research Center reports [18, 19]. For each country that lacked survey data for the percentage of circumcised males, the prevalence of MC was estimated from the sum of the numbers of Jewish and Muslim males. We assumed that 99.9 % of these, but none of non-Jewish and non-Muslim males, were circumcised. This compares with an assumption of 100 % used in the 2007 WHO estimates [15].

Circumcision of males as part of “coming of age” rituals is common in a substantial proportion of countries in eastern and southern Africa [17, 21], thus contributing to the high proportion of circumcised males found by self-report in DHS and AIS in those countries. Similarly, in many Pacific Island countries, MC is a cultural practice that forms part of traditional “coming of age” ceremonies of the indigenous Polynesian population [21–24]. Since the statistics on MC in Polynesian countries are not well-documented, estimates of MC prevalence in these countries were based on the proportion of people in each country who are indigenous, as ascertained by Internet searches by the name of the country and population category. In countries lacking survey data, but with high adherence to cultural customs concerning MC, the proportion of circumcised indigenous males was assumed to approximate 95 %. Since the total population of all Pacific Island nations represents about 0.1 % of the world’s population [25], data for Pacific Island countries made little contribution to our estimate of global MC prevalence.

Since MC is performed worldwide to treat adverse medical conditions such as phimosis, paraphimosis, balanoposthitis, and penile cancer [4, 26–28], no country is likely to have a MC prevalence of zero. We therefore set the lowest estimate for any country at 0.1 %, which we regard as conservative. In Denmark, for example, where non-medical circumcision is rare, a large survey found 4.5 % of Lutheran and non-religious men were circumcised [29]. Most of these MCs took place after infancy and, given historical opposition to MC in Denmark, were probably for treatment of an adverse medical condition caused by the presence of the foreskin [29]. In Australia, where MC has been common in infancy for many years, of the 11.5 % of circumcised men in one study who had been circumcised after infancy, the main reasons were phimosis (43 %) and parental wishes (40 %) [30].

### Estimation of global prevalence

In order to estimate the global prevalence of circumcision, we started with known figures for number of males in each country using two different authorities due to limitations in various databanks. We used CIA data for the total population of each country in the world [25] and data for sex ratio [31]. The total male population for each country was then determined using the formula a/(a + 1) x b, where "a" is the ratio of males-to-females in a given country [31] and "b" is the total population of that country [25]. For six very small countries with populations between 48 and 2210 (Christmas Island, Niue, Norfolk Island, Pitcairn Island, Svalbard, and Tokelauno) no data were available, so the average sex ratio for the entire world of 1.014 males per female [31] was used instead in our calculations. The second source, UN data for males aged 15–64 years [32], provides the age range used by most surveys that estimate MC prevalence. This database, however, lacks information for 45 countries and territories, many of which are small. Dividing the number of circumcised males by the total number of males in a country gave figures for the fraction of circumcised males in each country.

We then summed the number of males in each country to obtain the total number of males in the world. Following this, we summed the number of circumcised males in each country to obtain a total number of circumcised males globally. Dividing the latter by the former yielded an estimate of the percentage of males globally who are circumcised. Calculation of 95 % CI used the equation σ ± 1.96 ÷ square root of n, where x is the mean, σ is the standard deviation, n is the sample size, and the confidence coefficient is 1.96, and Excel was used to calculate the margin of error, as well as the upper and lower bound.

## Results

### Data obtained from surveys

Additional file 1 lists alphabetically those countries for which surveys of MC prevalence were available [26, 27, 29, 33–163] and provides reported country estimates, together with size of the survey, age range of males, national or regional survey scope, urban or rural and other demographic information, and the relevant citation.

### Estimates for all countries

The estimated percentage of circumcised males for each country or territory appears in Additional file 2, column 4. Nationally representative survey data in this file had the benefit of involving large numbers, thus increasing the precision of the estimates. However, self-report tends to introduce uncertainty because manhood initiation ceremonies in some traditional settings of sub-Saharan Africa may or may not include complete MC [164]. Since most of the values obtained were for males aged 15 years of age and over, they reflected MCs that had been performed in infancy or by late adolescence, depending on country and culture. Purported MC estimates in infancy may be unreliable because they are based on hospital discharge data and many boys are circumcised after discharge of the mother and her baby post-partum, whereas survey data in mature males appear to be more accurate [4]. In the last column of Additional file 2, “Survey” indicates that the value shown was obtained from survey data. This file also shows the percentage of Muslims in each country (column 2) and the percentage of Jews (column 3) [33]. In instances in which cultural traditions in the form of “coming of age” rituals were used as a basis for the estimate, this is indicated as “Culture” in the last column of Additional file 2. “Culture” was also used to indicate a country in which culture does not traditionally support MC.

Additional file 2, column 6, shows the total number of circumcised males in each of the 237 countries and territories, calculated from CIA databases as described in the Methods section. For simplicity, raw values generated by our computations are shown. These are overly precise and should not be taken literally as they are merely working numbers suitable for use in further calculations. While 95 % CI would help show the degree of accuracy of the estimates, these were not available for most countries. The 95 % CI for estimated prevalence of MC in the USA was ±2.5 % of the mean [132]. It is likely that 95 % CI for other countries may be wider than these.

Since CIA data does not give population figures by age group, we used the UN database of number of males aged 15–64 [32] to determine MC prevalence for a limited number of countries (Additional file 3).

Table 1 summarizes the percentage of MC prevalence determined for each country and territory in the world.

### Estimation of global prevalence

We then summed the total number for each country, starting first with figures obtained using the CIA database to calculate total males. This yielded an overly precise working figure of 1,412,252,836 circumcised males among the total number of 3,654,384,123 males in the world, constituting a working value for the purposes of the next calculation. Simple division of the former by the latter figure estimated global MC prevalence at approximately 38.65 % (95 % CI: 33.7, 44.4). Prevalence estimates were based on religion for 136 countries (57.4 %), survey data for 73 countries (30.8 %), and culture for 20 countries (8.4 %). In only seven countries (2.9 %) was no information available. In countries with survey data on MC prevalence, the total number of males was 2,694,086,787, i.e., 73.7 % of the total global male population. The percentage of circumcised males in these countries was 33.2 % overall. In 63 countries (26.6 %), adult MC prevalence exceeded 90 %. MC prevalence was greater than the global average of 36.7–38.7 % in 95 countries (40.1 %) and lower in 142 (59.9 %) countries.

Given that 23.2 % (1.6 billion) of the total population in the world is Muslim [18] and 0.18 % is Jewish [20], the proportion of males circumcised for religious reasons globally would be approximately 62.1 %. The rest would have been circumcised for reasons such as individual and family preference, which could include, for example, Christian faith, depending on Biblical interpretation by individuals, medical indications, cultural reasons such as “coming of age” ceremonies, or as part of HIV prevention programs in sub-Saharan African countries experiencing epidemics of heterosexual HIV transmission.

Based on UN data for number of males aged 15–64 as the denominator in our calculation of MC prevalence (Additional file 3), we obtained a figure of 36.7 % (95 % CI: 31.4, 42.0).

## Discussion

The true global MC prevalence is not known precisely and can only be estimated. Nevertheless, some estimates are more reliable than others. Global MC prevalence was asserted to be 20 % by Wallerstein in 1985 [165], but no sources or methodology were presented. Similarly, Williams & Kapila [166] and Hutcheson [167] estimated a prevalence of one in six, although neither article provided a basis for the estimate.

The earliest attempt to obtain a somewhat systematic estimate concluded that 23 % of males globally were circumcised [168]. That estimate, based on data through 1994, had some serious deficiencies, not least of which was the assumption, which was acknowledged as a limitation, that only Jews, Muslims, North Americans, and "Tribal Religionists" in Africa were circumcised. There was also poor consideration of populations falling into multiple groups; for example, North American Muslims appeared to have been counted twice.

The WHO published online a better attempt to estimate global prevalence in 2007 [15]. This analysis utilized global numbers of Muslims and Jews as a base, and then added MC prevalence of the non-Muslim, non-Jewish populations of 17 countries. From the resulting total, global prevalence was calculated as 30 %. A weakness of the approach was that MC prevalence data were likely gathered from a population sample that included Muslims and Jews, resulting in a probable overestimate when applied to the smaller non-Muslim, non-Jewish population. A second weakness was the relatively small number of countries for which non-religious MCs were considered. The increasing proportion of Muslims in the world population over time [19], as well as on-going VMMC programs in sub-Saharan Africa, might account in part for our higher estimate of 37–39 % for global MC prevalence compared to the 30 % reported in 2007 by the WHO based on an incomplete set of countries globally [7]. Additionally, since 2007 there has been a substantial increase in the availability of survey data, such as those compiled by DHS, AIS, and other reputable bodies, as well as publications in peer-reviewed journals, resulting in a stronger information base. A 34 % increase in infant MC in Germany between 2008 and 2011 could have been from a rise in the Muslim population in this traditionally non-circumcising country or perhaps other factors [169]. A disproportionate rise in Muslim immigrants as a proportion of the total population of other countries would contribute to increases in MC prevalence in each.

While the present study involved the most thorough analysis yet conducted, several limitations should be noted. All studies to date have suffered from the general difficulty of estimating MC prevalence in the absence of national surveys for every country. This may, however, be improving because DHS and AIS have increased their inclusion of questions on self-reported MC status. Survey data are based on the subpopulation of the total population that participated in the survey. Uncertainty could be of the order of 5–10 %. The 95 % CI for US estimates by the CDC were ±2.5 % of the mean, indicative of a high degree of precision in that study [132]. Large nationally representative surveys were more likely to provide the most accurate estimates of MC prevalence, whereas small surveys in sub-regions of a country have the potential to generate MC prevalence data that deviate from the actual national figure. As an example, a more recent, larger national survey in Brazil found overall MC prevalence to be 1.3 % [48], noting higher figures for cities in the south compared with rural areas in the north of this country. This demonstrated that MC prevalence figures from older surveys confined to Sao Paulo [50–52] and Rio de Janeiro [49] had yielded figures higher than the national average.

As well as significant regional variation in MC prevalence in some surveys, there was a higher prevalence noted among individuals and families with higher education and income.

Our estimates for MC prevalence in many smaller countries and territories tended to be less precise. Since those countries were small, however, the estimates for them made little contribution to our estimate of global MC prevalence. We chose, however, not to group these separately because we expected some readers would be interested in seeing estimates for these individual countries or territories.

Because CIA data only allowed us to estimate the total number of males in each country, whereas most surveys we used gave estimates of MC prevalence for males aged ≥15 years, our estimates for the total number of circumcised males when using CIA data for total males applied MC estimates to the total number of males regardless of age. Using this approach, we assumed, however, that if MC was performed it would have taken place in most males by 15 years of age. We nevertheless recognize that MC incidence may be rising or falling in some countries. This represents an unavoidable potential limitation to estimates based on CIA data for number of males in each country.

We overcame this limitation by using UN estimates of males aged 15–64 in each country. But the drawback of the UN database was that it did not include 45 countries. Nevertheless, those countries that were included likely led us to generate more accurate MC estimates for those than when using CIA figures for total males.

As a consequence of the absence of MC survey data available for many countries, it was necessary to estimate MC prevalence in some countries on the basis of the prevalence of ethnic/religious groups known to perform it, most notably Muslims [18, 19] and Jews [20]. Estimating MC prevalence based on the prevalence of certain religions in some countries is not completely accurate because (i) uncircumcised Muslims and Jews do exist, so the proportion of circumcised males in these groups is less than 100 %, which is why we used a figure of 99.9 %, and (ii) a percentage (possibly 5–10 % in developed countries) of males are circumcised for medical reasons such as phimosis, paraphimosis, balanitis, or conservative treatment of early stage penile cancer [28]. In this regard, our use of a minimum of 0.1 % for MC prevalence in a country for which no survey data were available is likely to be an underestimate.

In relation to religion, examination of countries that had both survey data on proportion of Muslims who were circumcised and data on the Muslim population proportion showed a close match: Comoros (99.4 % vs. 98.2 %, respectively), Gambia (90.0–99.0 % vs. 95.3 %), Guinea (96.0 % vs. 84.2 %), Indonesia (92.5 % vs. 88.1 %), Mali (86.0 % vs. 92.4 %), Mauritania (77.0 % vs. 99.2 %), Niger (92.0–99.0 % vs. 98.3 %), Sierra Leone (96.1 % vs. 71.5 %), and Somalia (93.0–94.0 % vs. 98.6 %). The one exception was Albania, where survey data suggested 47.7 % of males were circumcised but 77.9 % of the population was Muslim [33]. An AIDS Indicator Survey of Uganda determined the prevalence of MC in Muslim men and found 99.6 % were circumcised [148]. Data for other countries show, with few exceptions, a MC prevalence of 95–100 % for Muslims. In Albania, and possibly other countries, it is possible that a proportion of Muslims remained uncircumcised, that MC had not taken place by the age of 15 years, or there might have been reporting bias. Survey data for the UK found 98.7 % of Jewish men were circumcised [151]. From available data, it seems that the bigger the Muslim or Jewish population in a country, the more closely the actual MC prevalence likely matches the percentage of Muslims or Jews. In contrast, in countries in which Muslim people are minorities, the proportion of Muslim males who are circumcised was 71–85 %. Perhaps societal or access factors in such countries may be influencing the decision by Muslim or Jewish parents to have their boy circumcised.

Cultural considerations are of interest, especially in the world’s most populous country, China, where circumcision is not generally part of the culture [170]. Prevalence in China varies by subregion and there is a growing Muslim population in China’s western provinces. Awareness of the health benefits may be rising, as judged by publications and research by Chinese investigators, as well as the development in China of MC devices such as the Shang Ring [171].

**Fig. 2:**
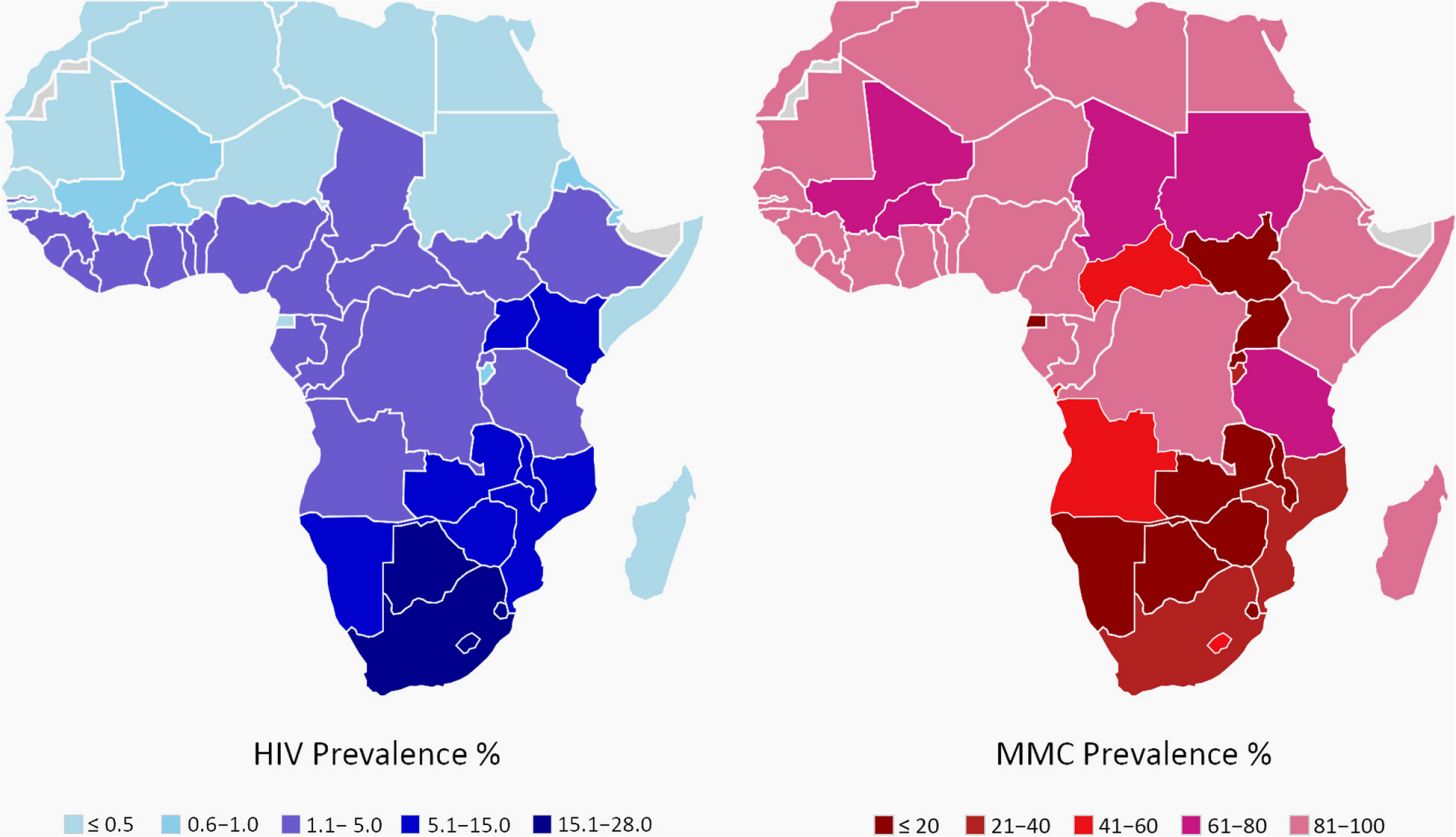
Map of HIV prevalence (left) and MC prevalence (right) for countries in the continent of Africa, where the main mode of HIV transmission is heterosexual intercourse

In sub-Saharan Africa, early recognition of the strong correlation between low MC prevalence and higher HIV prevalence prompted epidemiological studies to determine whether the two were linked. Confirmation of the causal link in several randomized controlled trials led to the promotion of MC for HIV prevention, with over 10 million having already undergone the procedure since 2009 [10]. Figure 2 uses the current data and the most recent country-specific HIV figures [9] to demonstrate this relationship in Africa.

Predicting MC prevalence based upon religion or culture is an approximation at best. Comparing countries for which both predicted and reported MC rates are available showed that reported MC prevalence generally exceeded religion-based predictions for these particular countries (Additional file 2). This may suggest that actual MC prevalence is greater than the value we estimated based on religion or culture in some countries lacking MC survey data. However, large surveys base MC prevalence on self-reports and even with the use of drawings or photographs to assist men in accurate self-reporting, some men who are not circumcised report that they are, while some who are circumcised report that they are not [94, 164, 172]. For 30.8 % of countries, MC prevalence was based on survey data rather than religion. The total number of men in these countries (2.7 billion) comprised 73.7 % of the global population of males. The proportion of males in surveyed countries who were circumcised was 33.2 %. Considering that there were 73 countries with survey data (30.8 %), but 136 (57.4 %) with data based on religion and 20 (8.4 %) based on culture, our estimate for global MC prevalence of 36.7–38.7 % would appear reasonable, particularly in light of possible underestimation of circumcisions in non-survey countries performed for medical reasons or personal preference.

## Conclusions

As accurately as might reasonably be expected, the present study has determined estimates for the prevalence of MC in every country in the world. Altogether, our findings suggest that MC prevalence globally is approximately 37.7 %, although the real percentage could be slightly higher or lower than this. Given the known benefits and low risks of MC [4] and recent affirmative recommendations in the interests of public health and disease prevention by the American Academy of Pediatrics, the CDC, the WHO and UNAIDS, as well as the large-scale roll out of VMMC by multilateral donors and agencies, the present findings on current prevalence of MC across geographies and cultures may help guide policy development and resource allocation in all countries.

## Additional files

Additional file 1:
**This spreadsheet provides data from published surveys on MC prevalence in all countries for which such studies have been conducted.** (XLSX 45 kb) Additional file 2:
**This spreadsheet shows data for number of males using CIA data and estimates of number of circumcised males in all 237 countries and territories in the world, as well as basis for the estimate and MC percentage for each.** (XLSX 32 kb) Additional file 3:
**This spreadsheet shows data for number of males using UN data for all but 45 countries and territories in the world and estimates of number of circumcised males in all but these 45, as well as basis for the estimate and MC percentage for each.** (XLSX 29 kb)
