# Erratum to: Estimation of country-specific and global prevalence of male circumcision

**DOI:** 10.1186/s12963-016-0080-6

**Published:** 2016-04-04

**Authors:** Brian J. Morris, Richard G. Wamai, Esther B. Henebeng, Aaron A. R. Tobian, Jeffrey D. Klausner, Joya Banerjee, Catherine A. Hankins

**Affiliations:** School of Medical Sciences and Bosch Institute, University of Sydney, Sydney, NSW 2006 Australia; Department of African-American Studies, Northeastern University, Boston, MA 02115 USA; College of Science, Northeastern University, Boston, MA 02115 USA; Department of Pathology, School of Medicine, Johns Hopkins University, Baltimore, MD 21287 USA; Division of Infectious Diseases and Program in Global Health, David Geffen School of Medicine, University of California Los Angeles (UCLA), Los Angeles, CA 90095 USA; Jhpiego, an affiliate of Johns Hopkins University, Washington, DC 20009 USA; Department of Global Health, Academic Medical Centre and Amsterdam Institute for Global Health and Development, University of Amsterdam, Amsterdam, 1105 AZ The Netherlands; Department of Infectious Disease Epidemiology, Faculty of Epidemiology and Population Health, London School of Hygiene and Tropical Medicine, London, WC1E 7HT UK

Following publication of the original article in Population Health Metrics [1], the authors wish to alert the reader to the following errors in male circumcision (MC) prevalence for 6 of the 237 countries and territories in Table 1.

**Table 1:** Percentage of circumcised males in each of the 237 countries and territories in the world*

Country/Territory	MC %	Country/Territory	MC %	Country/Territory	MC %
Afghanistan	99.8	Ghana	91.6	Oman	87.7
Albania	47.7	Gibraltar	6	Pakistan	96.4
Algeria	97.9	Greece	4.74	Palau	95
American Samoa	95	Greenland	0.1	Panama	0.95
Andorra	1.1	Grenada	0.3	Papua New Guinea	10.1
Angola	57.5	Guam	95	Paraguay	0.11
Anguilla	0.3	Guatemala	0.11	Peru	3.7
Antigua & Barbuda	0.6	Guernsey	0.1	Philippines	91.7
Argentina	2.9	Guinea	84.2	Pitcairn Islands	0.1
Armenia	0.1	Guinea-Bissau	93.3	Poland	0.11
Aruba	0.46	Guyana	12	Portugal	0.61
Australia	58	Haiti	6.2	Puerto Rico	0.14
Austria	5.8	Holy See (Vatican)	0.1	Qatar	77.5
Azerbaijan	98.5	Honduras	0.1	Romania	0.34
Bahamas, The	0.2	Hong Kong	28	Russia	11.8
Bahrain	81.2	Hungary	0.78	Rwanda	13.3
Bangladesh	93.2	Iceland	0.1	Saint Barthelemy	0.1
Barbados	0.9	India	13.5	Saint Helena, Ascens	0.1
Belarus	0.32	Indonesia	92.5	Saint Kitts & Nevis	0.3
Belgium	22.6	Iran	99.7	Saint Lucia	0.1
Belize	0.1	Iraq	98.9	Saint Martin & Tristan	0.1
Benin	92.9	Ireland	0.93	Saint Pierre & Miquel	0.2
Bermuda	0.8	Isle of Man	0.2	Saint Vincent & Grena	1.7
Bhutan	1.0	Israel	91.7	Samoa	95
Bolivia	0.11	Italy	2.6	San Marino	0.1
Bosnia & Herzegovina	41.6	Jamaica	14	Sao Tome & Principe	0.1
Botswana	15.1	Japan	9	Saudi Arabia	97.1
Brazil	1.3	Jersey	0.1	Senegal	93.5
British Virgin Islands	1.2	Jordan	98.8	Serbia	3.71
Brunei	51.9	Kazakhstan	56.4	Seychelles	1.1
Bulgaria	13.4	Kenya	91.2	Sierra Leone	96.1
Burkin Faso	88.3	Kiribati	0.1	Singapore	14.9
Burma	3.5	Korea, North	0.1	Sint Maarten	0.06
Burundi	61.7	Korea, South	77.0	Slovakia	0.15
Cabo Verde	0.1	Kosovo Islands	91.7	Slovenia	8.5
Cambodia	3.5	Kuwait	86.4	Solomon Islands	95
Cameroon	94	Kyrgyzstan	91.9	Somalia	93.5
Canada	31.9	Laos	0.1	South Africa	44.7
Cayman Islands	0.2	Latvia	0.38	South Sudan	23.6
Central African Republic	63.0	Lebanon	59.7	Spain	6.6
Chad	73.5	Lesotho	52	Sri Lanka	8.5
Chile	0.21	Liberia	97.7	Sudan	39.4
China	14.0	Libya	96.6	Suriname	15.9
Christmas Island	0.1	Liechtenstein	4.8	Svalbard	0.1
Cocos (Keeling)	95	Lithuania	0.2	Swaziland	8.2
Columbia	4.2	Luxembourg	2.42	Sweden	5.1
Comoros	99.4	Macau	0.1	Switzerland	5.9
Congo, Dem Rep	97.2	Macedonia	33.9	Syria	92.8
Congo, Republic	70	Madagascar	94.7	Taiwan	8.3
Cook Islands	95	Malawi	21.6	Tajikistan	99
Costa Rica	0.15	Malaysia	61.4	Tanzania	72
Cote d’Ivoire	96.7	Maldives	98.4	Thailand	11.9
Croatia	1.34	Mali	86	Timor-Leste	6.4
Cuba	0.11	Malta	0.3	Togo	95.2
Curacao	0.07	Marshall Islands	0.1	Tokelau	95
Cyprus	22.7	Mauritania	99.2	Tonga	95
Czech Republic	0.14	Mauritius	16.6	Trinidad & Tobago	5.8
Denmark	5.3	Mexico	15.4	Tunisia	99.8
Djibouti	96.5	Micronesia, Fed States	0.1	Turkey	98.6
Dominica	0.2	Moldova	0.5	Turkmenistan	93.4
Dominican Repub	13.7	Monaco	0.5	Turks & Caicos Is	0.1
Ecuador	0.11	Mongolia	4.4	Tuvalu	95
Egypt	94.7	Montenegro	18.5	Uganda	26.7
El Salvador	0.11	Montserrat	0.1	Ukraine	2.3
Equatorial Guinea	87	Morocco	99.9	United Arab Emirates	76
Eritrea	97.2	Mozambique	47.4	United Kingdom	20.7
Estonia	0.25	Namibia	25.5	United States	80.5
Ethiopia	92.2	Nauru	95	Uruguay	0.62
Falkland Islands	0.1	Nepal	4.2	Uzbekistan	96.5
Faroe Islands	0.1	Netherlands	5.7	Vanuatu	95
Fiji	55	New Caledonia	50	Venezuela	0.33
Finland	0.82	New Zealand	33.0	Vietnam	0.2
France	14	Nicaragua	0.1	Virgin Islands	0.55
French Polynesia	78	Niger	95.5	Wallis & Futuna	0.1
Gabon	99.2	Nigeria	98.9	West Bank	99.9
Gambia, The	94.5	Niue	95	Western Sahara	99.6
Gaza Strip	99.9	Norfolk Island	0.1	Yemen	99.0
Georgia	10. 6	Northern Mariana Is	90	Zambia	21.6
Germany	6.7	Norway	3.0	Zimbabwe	9.2

*The reader is referred to Additional files 1 and 2 in the supplementary material in order to understand the use of our methodology for deriving the values shown in this Table

–United States: The value for MC prevalence in the United States (US) is shown as 70.1, which is from reference 27, but this value should have been 80.5, as reported in reference 26. The figure of 70.1 is for 222,546 privately insured US males aged 1–18 years, whereas the figure of 80.5 is for 6,294 US males aged 14–59 years drawn from National Health and Nutrition Surveys by researchers at the US Centers for Disease Control and Prevention (CDC). The 80.5 figure is more representative of the overall male population in the US. As stated in the Methods section, circumcision prevalence figures for mature males across this general age range were used for all countries and territories when available. Only for Canada (infants) and Taiwan (boys aged 7–13 years) were the surveys that were available not for mature males.

–Germany: MC prevalence in Table 1 is shown as 10.9 %, but should have been 6.7 %. The former came from a nationwide survey of 8,985 males aged 1–17 (reference 82), whereas the latter is from a survey of 2,490 mature males aged 30–61 (Reference 83).

–Thailand: 11.9 % rather than 23.4 %, which arose from a calculation error.

–Australia: 85 % for males aged 16–64 (reference 36) rather than 26.6 %, which was the weighted average of these older males together with figures of 11 % for males aged 0–6 months (reference 37) and 10.9 % for newborn males (reference 38).

–Zambia: 21.6 % from 2014 data (reference 160) rather than 12.8 % from 2007 data (reference 161).

–Pitcairn Island: 0.1 % rather than 0 % owing to our decision to make 0.1 % the minimum for any country because MC is used commonly to treat foreskin-related medical conditions.

Use of these corrected figures led to estimates of 1,425,692,596 for total number of circumcised males globally based on 2015 CIA data for total males and 907,738,816 based on United Nations and CIA data. MC prevalence was calculated as 39.0 % (95 % CI: 33.7, 44.3) and 37.1 % (95 % CI: 31.8, 42.4), using 2015 CIA data for sex ratio and total population in 237 countries and territories globally and on 2015 United Nations figures for males aged 15–64 years, respectively.

The use of the corrected figures does not alter our rounded estimate for global MC prevalence of 37–39 %.

The corrected Table 1 appears below. Additional files 1, 2 and 3 referred to below are available for this article on-line.

## Additional files

Additional file 1: This spreadsheet provides data from published surveys on MC prevalence in all countries for which such studies have been conducted. (XLSX 49 kb) Additional file 2: This spreadsheet shows data for number of males using CIA data and estimates of number of circumcised males in all 237 countries and territories in the world, as well as basis for the estimate and MC percentage for each. (XLSX 29 kb) Additional file 3: This spreadsheet shows data for number of males using UN data for all but 45 countries and territories in the world and estimates of number of circumcised males in all but these 45, as well as basis for the estimate and MC percentage for each. (XLSX 31 kb)
